# COVID-19 and Thyroid: Progress and Prospects

**DOI:** 10.3390/ijerph17186630

**Published:** 2020-09-11

**Authors:** Francesca Gorini, Fabrizio Bianchi, Giorgio Iervasi

**Affiliations:** Institute of Clinical Physiology, National Research Council, 56124 Pisa, Italy; fabrizio.bianchi@ifc.cnr.it (F.B.); iervasi@ifc.cnr.it (G.I.)

**Keywords:** COVID-19, thyroid, subacute thyroiditis, thyrotoxicosis, thyroid disease

## Abstract

The outbreak of coronavirus disease 2019 (COVID-19), caused by severe acute respiratory syndrome coronavirus 2 (SARS-CoV-2), has rapidly spread worldwide. A number of serious effects on various organs and systems have been reported in humans, and recently emerging evidence on the potential association between the infection and thyroid dysfunction are attracting attention from the scientific community. This editorial critically summarizes the main findings on this topic published so far and defines research lines according to the translational approach from the bench to the bed to epidemiological studies and back again, aimed at patient care and effective public health measures.

## 1. Introduction

Among many correlated effects and comorbidities associated with coronavirus disease 2019 (COVID-19), those concerning the thyroid gland are also attracting increasing attention. This is not surprising as the thyroid hormones (THs) play a fundamental role in the regulation of multiple processes—development, growth, metabolism and energy supply—as well as in controlling the development of the nervous system during the early stages of life [1].

The COVID-19 comorbidities and multi-consequences are central on a clinical and public health level but also their relationships with perceptions and fears are not to be neglected [2].

It is also important to point out that the thyroid is functionally an organ “open” to environmental stimuli; in fact, it has been estimated that over 100 synthetic chemicals, released into the environment through various industrial activities, are able to negatively affect normal thyroid function [3].

The COVID-19 disease, caused by severe acute respiratory syndrome coronavirus 2 (SARS-CoV-2) infection, has rapidly spread since December last year and has led the World Health Organization to announce the COVID-19 outbreak as a pandemic on 11 March 2020. At the time of writing, according to data released daily by the COVID-19 Resource Center of Johns Hopkins University, the pandemic has affected over 23.5 million people in 188 countries causing over 810,000 deaths [4].

COVID-19, in addition to being strictly associated with the typical predisposing factors of infection, is linked to many individual and environmental risk factors, from age to non-compliance with distancing, from temperature conditions to air pollution concentrations [5,6]. The SARS-CoV-2 infection has the characteristic of being more aggressive or severe in susceptible subjects because of previous exposure to air pollution or the effect of comorbidities, some of which are typical of the COVID-19 clinical pattern [5,7,8]. Links with COVID-19 disease are also reported for the thyroid gland in addition to documentary ones with multiple environmental factors [9,10].

Serious and complex effects on various organs and systems of the human body including immune, circulatory, respiratory, digestive, hepatic, renal, and nervous systems, have been reported in patients with COVID-19 [11,12]. Some studies have also been conducted on reproductive functions with rather worrying results [13,14] and, more recently, preliminary results have been published on the effects on the thyroid gland.

In fact, some evidence had been gathered from the previous coronavirus pandemic, which began in 2002 and spread to 26 countries around the world. Among a group of 61 survivors of severe acute respiratory syndrome (SARS), with no pre-existing endocrine disease and studied three months after recovery, four (6.6%) were diagnosed with primary hypothyroidism [15], and thyroid lesions were found at autopsy in subjects who died of SARS [16].

## 2. COVID-19 and the Thyroid Gland

A first case-report of thyroid infection, known as subacute thyroiditis (SAT), a self-limiting inflammatory disease characterized by low-grade fever, neck pain, general malaise and thyroid gland dysfunction, following SARS-CoV-2 infection, was recently reported [17]. SAT is characterized by thyrotoxicosis of variable duration—usually weeks, more rarely months—followed by hypothyroidism and final recovery of the euthyroid condition [18]. This seems to be one of those situations in which even the description of a single case becomes relevant. The patient, a young woman who had tested positive for SARS-CoV-2 the previous month, had slightly increased heart rate and a painful, enlarged thyroid on palpation.

Laboratory tests had shown abnormalities of THs, such as increased free thyroxine (T4) and triiodothyronine (T3), suppression of thyrotropin (TSH), the appearance of anti-thyroglobulin antibodies and an increase in inflammatory markers and white blood cells, as well as an altered thyroid ultrasound image. The information that a month earlier, thyroid function and imaging were both normal is crucial. In addition, a complete regression of the initial symptoms and a significant improvement in both thyroid function and inflammatory markers were achieved within 2 weeks of starting treatment with prednisone.

The etiology and pathogenesis of SAT are not yet fully elucidated, but since a decade ago, the belief that the disease was triggered by a viral infection or post viral inflammatory reaction in genetically predisposed individuals had gained ground [19,20]. What was probably decisive in the formulation of the rationale and which is of great interest nowadays is the observation that SAT is generally preceded by an upper respiratory infection and occurs in association with the specific symptoms of viral pathology, and that many cases of SAT have been reported during viral outbreaks [20,21]. It should also be noted that many viruses, including mumps, rubella, adenovirus, orthomyxovirus, Epstein–Barr, hepatitis E, HIV, cytomegalovirus, and dengue fever, have been repeatedly related to the onset of SAT [20,22,23,24].

More recently, in a retrospective study conducted on 50 Chinese individuals with no previous history of thyroid diseases, hospitalized between January and March 2020 as affected by COVID-19, serum TSH levels were below normal values in 56% of patients. Concentrations of TSH and total T3 were significantly lower than those measured in both healthy subjects and in subjects with pneumonia of comparable degrees of severity but not for COVID-19 [25]. Furthermore, the decrease in TSH and total T3 levels was positively correlated with the severity of COVID-19. On the other hand, the total T4 concentration of individuals with COVID-19 did not differ significantly from the control group. Patients with COVID-19, while not receiving any thyroid hormone replacement therapy, had no significant difference in TSH as well as free and total T3 and T4 content with the control groups after recovery [25]. These results, although they should be interpreted with caution, especially due to the small size of the case series, lead to the hypothesis that COVID-19 could act on TSH-secreting cells, an effect that can be caused through direct action on pituitary cells or through systemic changes indirectly mediated by the activation of pro-inflammatory cytokines, by chronic hypoxic stress or by the treatment of patients with glucocorticoids. It is important to point out that low T3 levels are an indicator of non-thyroidal illness syndrome, a syndrome induced by a reduced conversion of T4 to T3 [26], and observed in all conditions of severe commitment of the whole organism and probably triggered by the same factors that cause a reduction in TSH (increase in cytokines and other inflammatory factors).

In Italy, in a recently published study, subjects admitted to high intensity of care units in 2020 due to COVID-19 (HICU-20), were compared with patients admitted to the same HICU in 2019 and therefore negative for SARS-CoV-2 (HICU-19) about the prevalence of thyrotoxicosis, suggestive for subacute thyroiditis [27]. Thyroid function at admission was then assessed in 93 consecutive patients in the HICU-20 group and in 101 consecutive subjects in the HICU-19 group. Fifty-two individuals with COVID-19 admitted to low intensity of care units (LHICU-20) were also studied. Excluding subjects with pre-existing thyroid disease, 15% of patients in the HICU-20 group had thyrotoxicity, and this was found in only 1% and 2% of patients in the HICU-19 and LHICU-20 groups, respectively, while of the 14 patients with thyrotoxicosis and COVID-19, the majority (64%) were men. Patients in the HICU-20 group also had a lower prevalence of pre-existing, both autoimmune and non-autoimmune, thyroid disease than those in the HICU-19 group, indicating that such conditions are not a risk factor for SARS-CoV-2 infection or for the degree of severity of COVID-19. Additionally, the subjects of the HICU-20 group had significantly lower TSH levels than those of the two other groups, whereas T4 concentrations were significantly higher only than the LHICU-20 group but not the HICU-19 group. On the other hand, no significant differences were found in the levels of free T3. Eight patients with COVID-19 and free of thyroid dysfunction at the time of admission were followed up for approximately 55 days after hospital discharge following a negative test for SARS-CoV-2. Two patients presented hypothyroidism and thyroid ultrasound features in line with autoimmune thyroiditis, while for the remaining six individuals, thyroid function returned to normal. Compared to that described by Chen et al. [26], the subjects with a high degree of severity of COVID-19, did not have the typical characteristics of patients with classic subacute thyroiditis, i.e., extremely low or even undetectable TSH values, high concentrations of T4, neck pain and leukocytosis, but, conversely, they displayed leukopenia. Overall, the study indicates that a non-negligible number of patients, requiring high intensity of care, present with thyrotoxicosis and low TSH concentrations, in line with a SARS-CoV-2-induced SAT and in a setting of non-thyroidal illness syndrome. The authors conclude by suggesting a routine evaluation of thyroid function in subjects with COVID-19 who require high intensity care, as they may frequently experience thyrotoxicosis caused by a SARS-CoV-2-related form of subacute thyroiditis [27].

It should also be highlighted that antithyroid drugs, used to inhibit thyroid activity in the case of hyperthyroidism, while not increasing the risk of COVID-19 infection or the probability of developing a more serious disease, can give rise to neutropenia in susceptible individuals who experience symptoms (sore throat, mouth ulceration, fever and flu-like illness) similar to the symptoms of COVID-19 (fever, new continuing cough and flu-like illness), making it difficult, if not impossible, for physicians to distinguish between these two diagnoses [28,29]. Since neutropenia induced by antithyroid drugs is instead associated with an increased risk of infections, this adverse effect, albeit rare, may favor the progression of COVID-19, reasonably through a reduction of the innate immune response or a generalized suppression of the immune system [28]. Moreover, classic antithyroid drugs such as methimazole or bromazole are not indicated for SAT treatment as SAT etiopathogenesis is different from the classic hyperthyroidism, being a consequence of the dismissal of THs into the circulation and not of the increased synthesis of the hormone on which the antithyroid drugs actually act.

So far the data available do not allow us to state that thyroid diseases represent a risk factor for the development of COVID-19 and, similarly, a higher prevalence of thyroid disease in patients with COVID-19 has not been detected. Nonetheless, a simple and low-cost thyroid evaluation accompanied by the administration of a questionnaire with specific sections on exposure to potential endocrine disruptor chemicals at hospital admission with subsequent follow-up could be useful for different goals: firstly, in patients with multiple diseases to avoid thyrotoxicosis first and thereafter hypothyroidism deriving from SAT, which may result in a worse general picture and may affect the metabolism of the drugs used for COVID-19 with altered therapeutic responses; moreover, to collect data for environmental epidemiology studies. Properly designed future studies will have the task of strengthening or weakening the emerging hypothesis or generating others.

## References

[1] Gorini F., Bustaffa E., Coi A., Iervasi G., Bianchi F. (2020). Bisphenols as Environmental Triggers of Thyroid Dysfunction: Clues and Evidence. Int. J. Environ. Res. Public Health.

[2] Cori L., Bianchi F., Cadum E., Anthonj C. (2020). Risk Perception and COVID-19. Int. J. Environ. Res. Public Health.

[3] European Parliament, Policy Department for Citizens’ Rights and Constitutional Affairs, 2019. Endocrine Disruptors: From Scientific Evidence to Human Health Protection. https://www.europarl.europa.eu/RegData/etudes/STUD/2019/608866/IPOL_STU(2019)608866_EN.pdf.

[4] John Hopkins University of Medicine Coronavirus Research Center. https://coronavirus.jhu.edu/map.html.

[5] Asfahan S., Deokar K., Dutt N., Niwas R., Jain P., Agarwal M. (2020). Extrapolation of mortality in COVID-19: Exploring the role of age, sex, co-morbidities and health-care related occupation. Monaldi Arch. Chest Dis..

[6] Ma Y., Zhao Y., Liu J., He X., Wang B., Fu S., Yan J., Niu J., Zhou J., Luo B. (2020). Effects of temperature variation and humidity on the death of COVID-19 in Wuhan, China. Sci. Total Environ..

[7] Comunian S., Dongo D., Milani C., Palestini P. (2020). Air Pollution and Covid-19: The Role of Particulate Matter in the Spread and Increase of Covid-19′s Morbidity and Mortality. Int. J. Environ. Res. Public Health.

[8] Copat C., Cristaldi A., Fiore M., Grasso A., Zuccarello P., Signorelli S.S., Conti G.O., Ferrante M. (2020). The role of air pollution (PM and NO2) in COVID-19 spread and lethality: A systematic review. Environ. Res..

[9] Nettore I.C., Colao A., Macchia P.E. (2018). Nutritional and Environmental Factors in Thyroid Carcinogenesis. Int. J. Environ. Res. Public Health.

[10] Fiore M., Conti G.O., Caltabiano R., Buffone A., Zuccarello P., Cormaci L., Cannizzaro M.A., Ferrante M. (2019). Role of Emerging Environmental Risk Factors in Thyroid Cancer: A Brief Review. Int. J. Environ. Res. Public Health.

[11] Huang C., Wang Y., Li X., Ren L., Zhao J., Hu Y., Zhang L., Fan G., Xu J., Gu X. (2020). Clinical features of patients infected with 2019 novel coronavirus in Wuhan, China. Lancet.

[12] Li Y., He F., Zhou N., Wei J., Ding Z., Wang L., Chen P., Guo S., Zhang B., Wan X. (2020). Multidisciplinary Team for COVID-19. Organ function support in patients with coronavirus disease 2019: Tongji experience. Front. Med..

[13] Jing Y., Run-Qian L., Hao-Ran W., Hao-Ran C., Ya-Bin L., Yang G., Fei C. (2020). Potential influence of COVID-19/ACE2 on the female reproductive system. Mol. Hum. Reprod..

[14] Segars J., Katler Q., McQueen D.B., Kotlyar A., Glenn T., Knight Z., Feinberg E.C., Taylor H.S., Toner J.P., Kawwass J.F. (2020). Prior and novel coronaviruses, Coronavirus Disease 2019 (COVID-19), and human reproduction: What is known?. Fertil. Steril..

[15] Leow M.K., Kwek D.S., Ng A.W., Ong K.C., Kaw G.J., Lee L.S. (2005). Hypocortisolism in survivors of severe acute respiratory syndrome (SARS). Clin. Endocrinol..

[16] Wei L., Sun S., Xu C.H., Zhang J., Xu J., Zhu H., Peh S.-C., Korteweg C., McNutt M.A., Gu J. (2007). Pathology of the thyroid in severe acute respiratory syndrome. Hum. Pathol..

[17] Brancatella A., Ricci D., Viola N., Sgrò D., Santini F., Latrofa F. (2020). Subacute Thyroiditis After Sars-COV-2 Infection. J. Clin. Endocrinol. Metab..

[18] Guimarães V.C., Jameson J.L., De Groot L.J. (2016). Subacute and riedel’s thyroiditis. Endocrinology Adult and Pediatric.

[19] Nishihara E., Ohye H., Amino N., Takata K., Arishima T., Kudo T., Ito M., Kubota S., Fukata S., Miyauchi A. (2008). Clinical characteristics of 852 patients with subacute thyroiditis before treatment. Intern. Med..

[20] Desailloud R., Hober D. (2009). Viruses and thyroiditis: An update. Virol J..

[21] Benbassat C.A., Olchovsky D., Tsvetov G., Shimon I. (2007). Subacute thyroiditis: Clinical characteristics and treatment outcome in fifty-six consecutive patients diagnosed between 1999 and 2005. J. Endocrinol. Investig..

[22] Bouillet B., Petit J.M., Piroth L., Duong M., Bourg J.B. (2009). A case of subacute thyroiditis associated with primary HIV infection. Am. J. Med..

[23] Assir M.Z., Jawa A., Ahmed H.I. (2012). Expanded dengue syndrome: Subacute thyroiditis and intracerebral hemorrhage. BMC Infect. Dis..

[24] Martínez-Artola Y., Poncino D., García M.L., Munné M.S., González J., García D.S. (2015). Acute hepatitis E virus infection and association with a subacute thyroiditis. Ann. Hepatol..

[25] Chen M., Zhou W., Xu W. (2020). Thyroid Function Analysis in 50 Patients with COVID-19: A Retrospective Study. Thyroid.

[26] Fliers E., Bianco A.C., Langouche L., Boelen A. (2015). Thyroid function in critically ill patients. Lancet Diabetes Endocrinol..

[27] Muller I., Cannavaro D., Dazzi D., Covelli D., Mantovani G., Muscatello A., Ferrante E., Orsi E., Resi V., Longari V. (2020). SARS-CoV-2-related atypical thyroiditis. Lancet Diabetes Endocrinol..

[28] Boelaert K., Visser W.E., Taylor P.N., Moran C., Léger J., Persani L. (2020). Endocrinology in the Time of COVID-19: Management of hyperthyroidism and hypothyroidism. Eur. J. Endocrinol..

[29] Dworakowska D., Grossman A.B. (2020). Thyroid disease in the time of COVID-19. Endocrine.

